# Correction: Laboratory strains of *Aedes aegypti* are competent to Brazilian Zika virus

**DOI:** 10.1371/journal.pone.0174081

**Published:** 2017-03-13

**Authors:** André Luis Costa-da-Silva, Rafaella Sayuri Ioshino, Helena Rocha Corrêa de Araújo, Bianca Burini Kojin, Paolo Marinho de Andrade Zanotto, Danielle Bruna Leal Oliveira, Stella Rezende Melo, Edison Luiz Durigon, Margareth Lara Capurro

The following information is missing from the Funding section: This study was supported by the National Institutes of Health/National Institute of Allergy and Infectious Diseases (NIH/NIAID) UO1 AI115595 (https://www.nih.gov/).
