# Supplementary figures and images for: Laboratory strains of Aedes aegypti are competent to Brazilian Zika virus
Source: PLoS One. 2017 Feb 10;12(2):e0171951. doi: 10.1371/journal.pone.0171951 (PMC5302382; doi:10.1371/journal.pone.0171951)

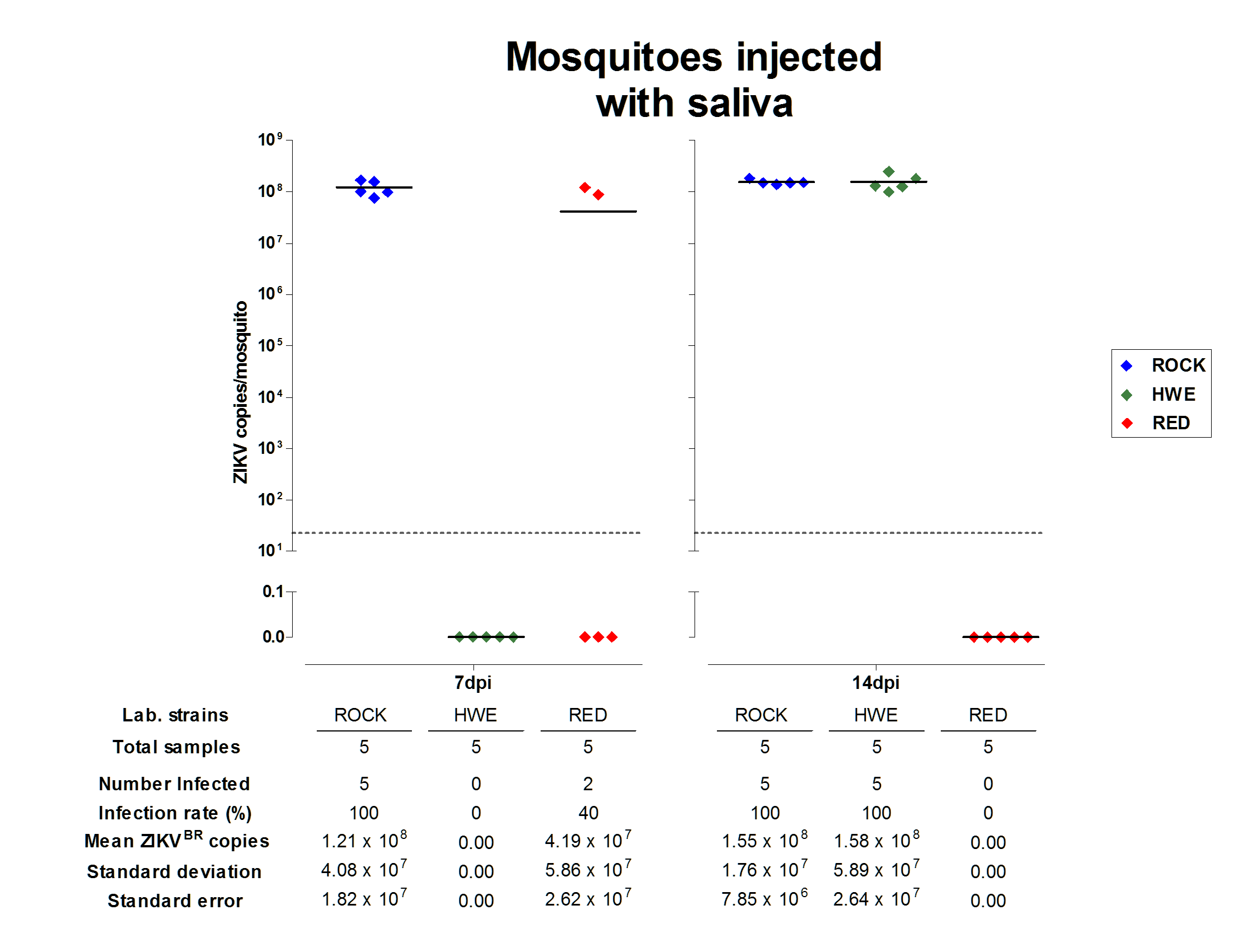

Supplement: S1 Fig — The prevalence and viral levels per mosquito were recorded after 11 days post-injection of saliva pools collected from ROCK, HWE and RED at 7 and 14 dpi. Each mosquito is represented by a solid diamond. Black bars indicate the mean viral copy numbers and the dashed grey line demonstrates the detection limit. (TIF) [file pone.0171951.s001.tif]
